# Radiography education with VR using head mounted display: proficiency evaluation by rubric method

**DOI:** 10.1186/s12909-022-03645-8

**Published:** 2022-07-28

**Authors:** Kengo Kato, Daisuke Kon, Teruo Ito, Shigeji Ichikawa, Katsuhiko Ueda, Yoshihiro Kuroda

**Affiliations:** 1 International University of Health and Welfare, Narita, Japan; 2 University of Tsukuba, Tsukuba, Japan

**Keywords:** Virtual reality, Simulation education, Head mounted display, Radiology technologist, Rubric, Self-learning

## Abstract

**Background:**

The use of head mounted display (HMD)-based immersive virtual reality (VR) coaching systems (HMD-VRC) is expected to be effective for skill acquisition in radiography. The usefulness of HMD-VRC has been reported in many previous studies. However, previous studies have evaluated the effectiveness of HMD-VRC only through questionnaires. HMD-VRC has difficulties in palpation and patient interaction compared to real-world training. It is expected that these issues will have an impact on proficiency. The purpose of this study is to determine the impact of VR constraints in HMD-VRC, especially palpation and patient interaction, on radiographic skills proficiency in a real-world setting.

**Methods:**

First-year students (*n* = 30) at a training school for radiology technologists in Japan were randomly divided into two groups, one using HMD-VRC (HMD-VRC group) and the other practicing with conventional physical equipment (RP group) and trained for approximately one hour. The teachers then evaluated the students for proficiency using a rubric method.

**Results:**

In this study, it was found that some skills in the HMD-VRC group were equivalent to those of the RP group and some were significantly lower than those of the RP group. There was a significant decrease in proficiency in skills related to palpation and patient interaction.

**Conclusions:**

This study suggests that HMD-VRC can be less effective than real-world training in radiographic techniques, which require palpation and patient interaction. For effective training, it is important to objectively evaluate proficiency in the real world, even for HMD-VRC with new technologies, such as haptic presentation and VR patient interaction.

**Trial registration:**

The study was conducted with the approval of the Ethics Committee of International University of Health and Welfare (Approval No.21-Im-035, Registration date: September 28, 2021).

**Supplementary Information:**

The online version contains supplementary material available at 10.1186/s12909-022-03645-8.

## Background

The head mounted display (HMD)-based immersive virtual reality (VR) coaching system (HMD-VRC) incorporates a variety of skills training programs [1–6]. HMD-VRC can play an important role in radiology training of technologists because it allows them to obtain virtual X-ray images without a risk of exposing the trainee or patient to radiation. Furthermore, HMD-VRC is suitable for the realistic experience of tasks involving physical movements within a large VR space. Therefore, HMD-VRC has begun its application in radiography training, which is one of the tasks of radiology technologists [7, 8]. The usefulness of HMD-VRC has been reported in many studies, and it is expected to be used in many training schools for radiology technologists in the future.

On the other hand, previous studies have also identified several issues that the HMD-VRC has technological difficulties in palpation and patient interaction [7]. Radiography requires touching the skeleton from the body surface, which serves as a reference point for determining the area to be radiographed. Patient interaction includes the instruction from radiologists to patients and the conversation between them; the radiologist should therefore ensure proper radiography conditions while avoiding painful incidents and uncomfortable communications. These HMD-VRC issues related to palpation and patient interaction may affect proficiency in radiography. The effectiveness of HMD-VRC has been reported in subjective proficiency surveys using questionnaires. However, this is a subjective evaluation and may not accurately reflect the educational effectiveness of HMD-VRC compared to education using physical equipment.

Proficiency evaluation of advanced skills in radiography is a qualitative learning evaluation, and it is difficult to evaluate quantitatively with numerical values as in a written test. We focused on the rubric method as one of the solutions to this issue [9]. The rubric method can be quantified by setting the scoring criteria, and the proficiency of different learning methods can be compared. In addition, the rubric method is a tool for evaluating the quality of performance with little bias.

Previously, Gunn et al. [10] reported the learning effectiveness of radiography in a VR environment using rubric evaluation. As a result of the training, it was possible to acquire skills equal to or slightly better than those using the physical machine. However, their research differs significantly from our study in that they used a PC monitor rather than an HMD as the visual display. In addition, only basic radiography techniques were included as rubric evaluation items. Consequently, it does not include many items, such as those related to communication, which are important in clinical practice [11]. In addition, the study by Gunn et al. [10] evaluated proficiency only in hand and foot radiography. However, the required level of skill varies depending on the radiographed area. Therefore, it is necessary to investigate the radiographic proficiency for other inspection target areas.

O'Connor et al. [7] also reported on radiographic training using HMD-VRC. The study included a questionnaire, in which 58% of the experiment participants indicated that they enjoyed VR, and 94% would recommend the system. On the other hand, technical glitches, the inherent inability to touch patients, and lack of feedback about learning were also noted. It should also be noted that their study is limited to subjective evaluation of student proficiency through questionnaires.

As described above, HMD-VRC can play an important role in training radiology technologists because it allows them to obtain virtual images without the risk of exposing the trainee or patient to radiation. Furthermore, HMD-VRC is suitable for the realistic experience of tasks involving physical movements within a large VR space. On the other hand, HMD-VRC presents difficulties in palpation and patient interaction compared to real-world training. It is expected that these issues will have an impact on proficiency. However, proficiency in radiography with HMD-VRC has not been adequately evaluated in a real-world setting. To evaluate the skills required in clinical practice, an overall evaluation including items that are difficult to achieve with the HMD-VRC is needed but has not yet been reported.

The purpose of this study is to determine the impact of VR constraints in HMD-VRC, especially palpation and patient interaction, on radiographic skills proficiency in a real-world setting. For this purpose, first-year radiography trainees (i.e., students before learning the radiographic method in the class) receive a self-learning experience using HMD-VRC and a mock class using a physical device. After training, all participants in the VR training as well as the group trained with the physical device are evaluated in the real world, since the radiographic skills used in the clinical setting are important. Using a rubric evaluation with new evaluation criteria including HMD-VRC issues as the evaluation method, the proficiency level of each training is quantitatively evaluated for comparison and validation.

## Materials and methods

### HMD-VRC system requirements

The main features HMD-VRC should have to facilitate the learning of radiography are: (i) the ability to control the simulated patient and X-ray tube in a highly immersive VR space using the HMD; (ii) the ability to reflect the viewing position, direction, and hand movements in training by tracking the position of the HMD and hand controller; and (iii) the ability of the radiography acquisition images to accurately respond to changes in exposure dose (tube voltage, tube current, exposure time) and the position of the patient and X-ray tube. By using a system that meets the requirements, radiography techniques can be trained in a way that is similar to real-world practice without the use of radiation.

### Rubric table

As shown in Table 1, 12 representative skills were selected from clinical performance and a rubric table was created after preliminary discussions between four teachers. The 12 items were established for skills including equipment operation, patient positioning, process management, and communication. The rubric table uses a 4-point scale. A score of 0 was set as the pre-learning stage, 1 as the initial learning stage, 2 as the minor adjustment stage, and 3 as the clinical adaptation stage. The written description was added to the rubric to enable appropriate judgment for each element.

**Table 1:** Relationship between rubric evaluation items and representative issues of HMD-VRC compared to Role-play

	**Rubric evaluation items**	**Issues of HMD-VRC compared to Role-play**	
		**Palpate**	**Patient interaction**
#1	Collimation		
#2	Direction of the X-ray beam angle		
#3	Location and centering of the X-ray beam	✓	
#4	Patient position	✓	✓
#5	Correct selection of source image receptor distance		
#6	Positioning the X-ray image detector	✓	
#7	Side marker placement		
#8	Voice guidance		✓
#9	Exposure parameter selection		
#10	Closing the door during exposure		
#11	Process control and safety		✓
#12	Hospitality and concern for patient pain	✓	✓

For the rubric table, 12 skills were extracted through discussions among four teachers to evaluate the radiography skills required for clinical practice. The rubric table is a 4-point scale. Sentences were written to enable appropriate judgment for each element of the rubric table. This is the relationship between representative issues related to the issues of the HMD-VRC and the rubric evaluation items in this study

### Experimental procedure

The study was conducted with the approval of the Ethics Committee of International University of Health and Welfare (Approval No.21-Im-035). The participants were first-year students of the Department of Radiological Sciences at International University of Health and Welfare. Table 2 shows the characteristics of the students who participated in this study (12 males; 18 females). We researched their first semester GPA (Grade Point Average) score of the first year of college and other factors related to learning outcomes.

**Table 2:** Characteristics of participants

**Sex**	Males: 12 students(HMD-VRC group: 5 students) Females: 18 students(HMD-VRC group: 10 students)
Age	18.9 ± 0.4 years
Time from training to evaluation	HMD-VRC group: 6.6 ± 2.2 days RP group: 8.8 ± 3.0 days
Experience using VR in daily life	None: 80% Less than 10 times: 20% More than 10 times: 0%

The participant's gender, age, time from training to evaluation date, and experience using HMD-type VR devices

**Fig. 1:**
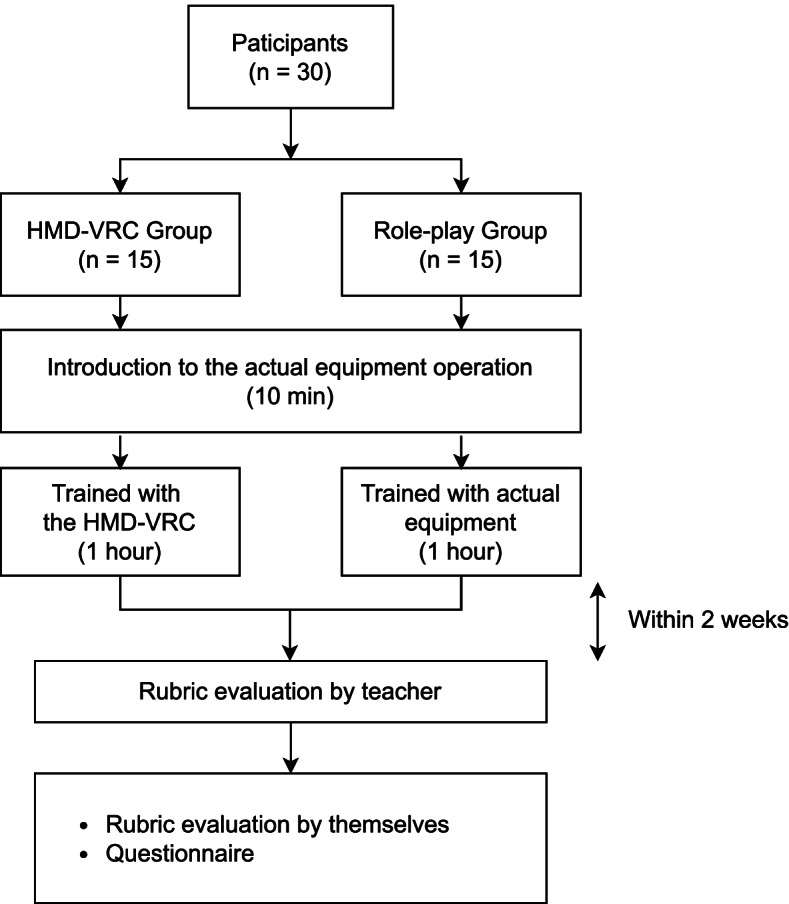
Experimental procedure. We used the 10 min before the start of the training to present the rubric table, explain the evaluation criteria and the physical equipment used for the evaluation. After the explanation, each group trained on the lateral elbow and PA chest radiography for about an hour. Then, within two weeks, the teachers conducted an evaluation using the rubric method on the students. After the evaluation was completed, the students evaluated their own proficiency using a rubric table. In addition, a questionnaire on learning was administered

Figure 1 shows the flow of the evaluation process. The participants were randomly divided into two groups. Education using HMD-VRC (HMD-VRC group, 15 students) and practical training using physical X-ray equipment (RP group, 15 students) were conducted. Randomization was performed by assigning random numbers to the students using a computer. The evaluation targets were lateral elbow and PA (postero-anterior) chest radiography. PA chest radiography is the most common area of radiography and also contains many important radiographic skills. Compared to hand radiography, there are many areas where the patient is instructed to hold the position, so care must be taken to avoid causing pain. In addition, since there are many important organs in the trunk, the exposure area must be carefully adjusted by palpation. Lateral elbow radiography is a typical joint radiography, in which the angle of incidence of radiation is adjusted by complex patient positioning to image the joint. Compared to PA chest and lateral elbow radiography, imaging to evaluate the hand or foot radiography are less complex patient positioning.

Study materials for lateral elbow and PA chest radiography were distributed before the training. We used 10 min before the start of the training to present the rubric table and to explain the evaluation criteria and the usage of the physical equipment. This was followed by approximately one hour of training in the lateral elbow and PA chest radiography.

**Fig. 2:**
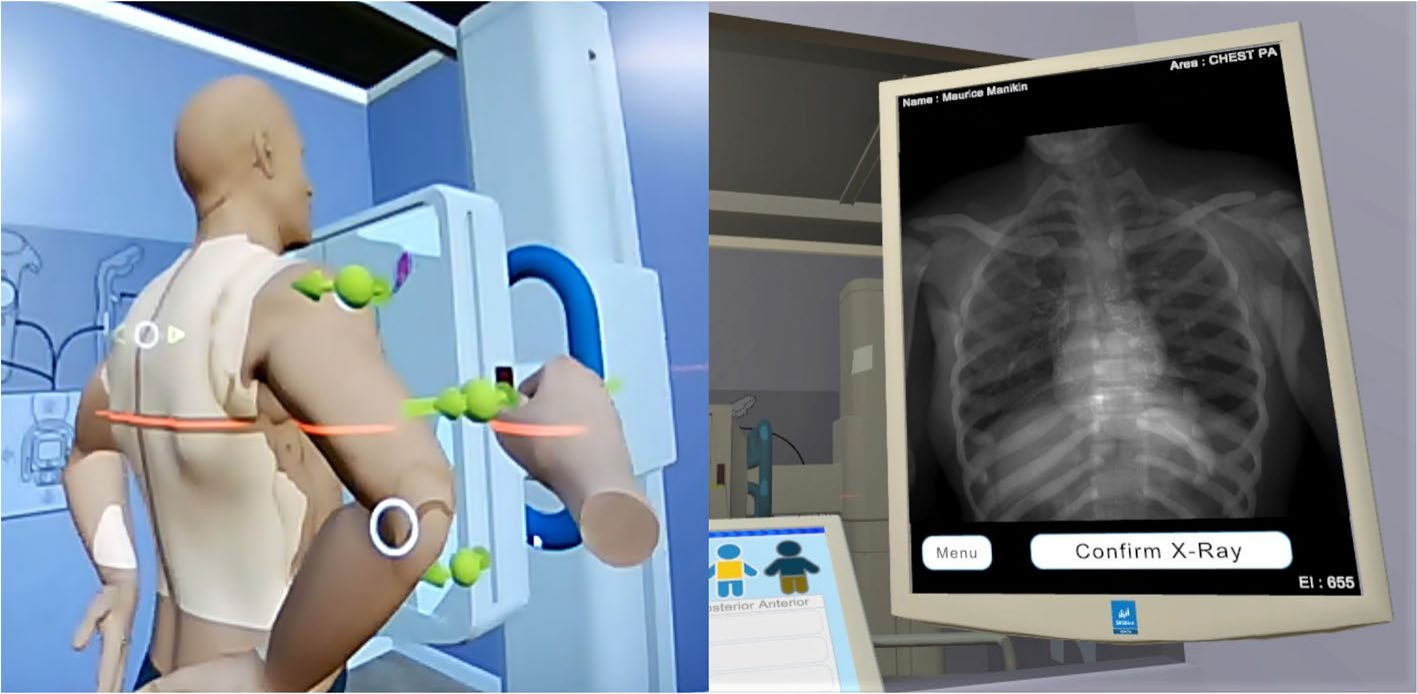
Captured image of patient positioning in VR X-Ray. Users can operate the X-ray equipment and the examinee in a highly immersive VR space. Users can also perform X-ray imaging in the VR space and train while checking the captured X-ray images

In the HMD-VRC group, one teacher was assigned to each student. The training using HMD-VRC was designed for self-learning. During the training, students taught themselves radiography procedures using materials and a detailed rubric evaluation table. The teachers did not evaluate the training but provided instruction in the basic use of the HMD-VRC. At this time, questions regarding the content of the reference materials were accepted, as beginning students lacked prerequisite knowledge and needed explanations of the training materials as appropriate. In this study, we used a VR X-Ray (manufactured by Skilitics and Virtual Medical Coaching, New Zealand; distributed by Siemens Healthcare GmbH, Germany) that met the system requirements for HMD-VRC. VR X-Ray uses the HTC VIVE headset and VIVE controller (HTC Corporation, Taiwan) as the HMD system, allowing users to learn various radiographic skills in a highly immersive VR space. As shown in Fig. 2, VR X-Ray allowed us to train radiographic techniques by manipulating simulated X-ray equipment and patient’s position in a highly immersive VR space. The patients simulated in HMD-VRC were adult males. The VR X-Ray does not give an evaluation to the student. A maximum 3 m × 4 m space can be prepared as a VR space, and users can move within it. The VR training steps include moving the X-ray equipment and patient in the VR space for correct positioning, then closing the door, determining the dose, and taking the virtual X-ray images. The student can evaluate the virtual x-ray image and repeat the radiographic procedure. HMD-VRC has the advantage that radiography training can be trained at the pace of one's own learning. On the other hand, HMD-VRC has difficulties in palpation and patient interaction compared to real-world training.

On the other hand, the RP group used X-ray equipment KXO-50G/DST-100A (TOSHIBA Corporation, Japan) and a human phantom PBU-60 (Kyoto Kagaku Corporation, Japan) for radiography. The teacher conducted a mock class, using an X-ray human phantom and a simulated patient for practice. To ensure that there was no variation in training time by the students, training was conducted in groups of up to two students.

Within two weeks after the training, the evaluation of proficiency using the physical equipment on one simulated patient was conducted. The two-week period was chosen to consolidate the evaluation days, although only about two people can be trained per day. Participants did not use the equipment from post-training to the time of evaluation. The evaluation using the rubric table was conducted by one teacher for each student. A total of four teachers conducted the evaluation, and discussions were held in advance to ensure that there were no discrepancies in the evaluation criteria. The teachers involved in creating and scoring this rubric have no specific training in VR or digital education but have more than 10 years of experience as radiology technologists working in clinical practice. In their clinical experience, they provided education to new employees and internship students. The evaluation was performed in the same order for all students: first the lateral elbow, and then the PA chest radiography. After the evaluation was completed, the students evaluated their own proficiency using a rubric table. In addition to administering the same questionnaire on learning to the HMD-VRC group and the RP group, the HMD-VRC group was additionally given a detailed questionnaire on HMD-VRC.

### Statistical analysis

R (version 4.1.2) was used as the statistical processing software. Comparisons between groups were made using the t-test for unequal variances without correspondence, with the significance level set at 5%. In the correlation analysis, the results were evaluated by Pearson's correlation coefficient. Correlation coefficients of |*r*|> 0.7 is highly correlated and |*r*|< 0.2 is uncorrelated. There were no cancellations or dropouts of participants. Errors by the participants in answering questions were considered to occur independently of the outcome and were excluded from the analysis data.

## Results

**Fig. 3:**
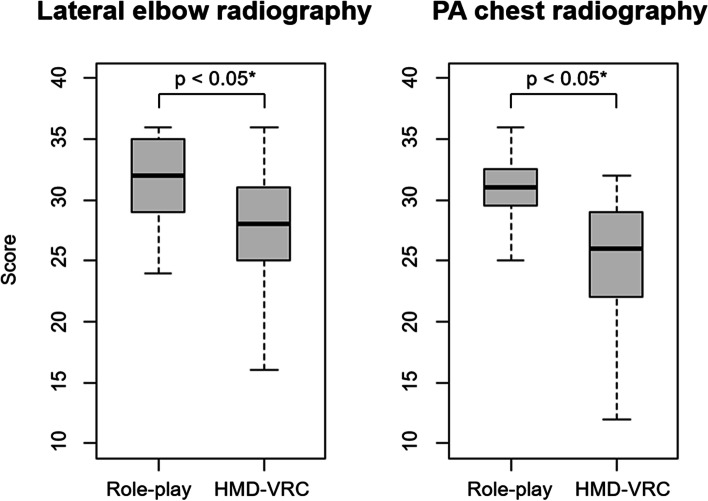
Total score using the rubric method administered to the students by the teachers. Box plot of the total evaluation scores in the rubric table when the students’ proficiency was evaluated by the teacher. The center line of the box plot indicates the median of the data. The top of the box indicates the third quartile and the bottom of the box indicates the first quartile. The upper whiskers and lower whiskers indicate the maximum and minimum total scores

**Fig. 4:**
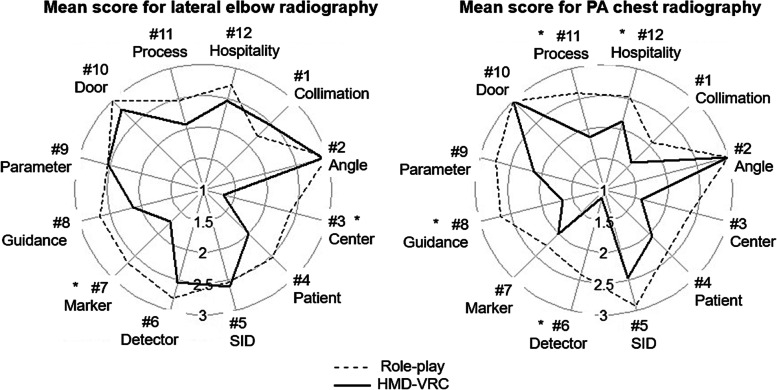
Evaluation of each skill in the HMD-VRC group and the RP group. The radar chart shows the average scores of the teachers’ evaluations of the students’ skills on the 12 items of the rubric table. Each individual was scored on a four-point scale from 0 to 3. The dotted line indicates the RP group, and the solid line indicates the HMD-VRC group. Each item corresponds to an item in Table 1. Compared to the HMD-VRC group, the RP group was rated significantly higher in #3 and 7 for lateral elbow and #6, 8, 11, and 12 for PA chest radiography

Figure 3 shows the proficiency of the HMD-VRC group was significantly lower than that of the RP group in lateral elbow and PA chest radiography. Figure 4 shows the average score of the itemized rubric evaluation given by the teachers to the students. Each individual was scored on a four-point scale from 0 to 3. The dotted line indicates the RP group, and the solid line indicates the HMD-VRC group. Each item corresponds to an item in Table 1. Some items (“Direction of the X-ray beam angle” and “Closing the door during exposure”) were comparable to those of the RP group, while others were significantly lower than those of the RP group (“Location and centering of the X-ray beam,” and “Side marker placement” for lateral elbow radiography and “Positioning the X-ray image detector,” “Voice guidance,” “Process control and safety,” and “Hospitality and concern for patient pain” for PA chest radiography). Additional file 1: Appendix 1 shows the results of a 5-point questionnaire (18 items) on the learning effectiveness of the HMD-VRC and RP groups. The questionnaire included surveys on “Improvement of radiographic techniques” and “State of motivation through education.” The questionnaire showed no significant differences between the HMD-VRC and RP groups. More than 70% of both the HMD-VRC and RP groups agreed with the question, “Do you feel that your radiographic skills have improved?” Similarly, more than 80% of both groups agreed with the question, “Were you motivated to learn?” Likewise, more than 90% of both groups agreed with the question, “Were you satisfied with the learning process?” In addition, more than 90% of both groups agreed with the question, “Are you interested in classes using VR?” The HMD-VRC group and the RP group did not differ significantly in the imaging time for lateral elbow and PA chest radiography.

The rubric evaluation scores for the students did not correlate with the first semester GPA score of the first year of college, experience using VR with HMD, or the time between the training date and the evaluation date. We also examined the frequency of playing computer games among HMD-VRC participants but found no correlation with their scores on the rubric evaluation.

As shown in Table 3, a questionnaire with five levels (Strongly Agree, Agree, Neither Agree nor Disagree, Disagree, and Strongly Disagree) was administered to 15 students using HMD-VRC, and the sum of “Strongly Agree” and “Agree” was expressed as “Agree,” and similarly, the sum of “Strongly Disagree” and “Disagree” was expressed as “Disagree” as a percentage. The results showed that the learning by HMD-VRC was favorably received. On the other hand, the percentages of “Agree” and “Disagree” were almost the same for the question, “Do you think you will be able to proceed with learning by using VR without teacher support next time?”.

**Table 3:** Questionnaire results for the students who used HMD-VRC

	Agree (%)	Disagree (%)	Neither (%)
● Before the research, do you have a positive impression of VR?	87	13	0
● After the research, do you have a favorable impression of VR?	93	0	7
● Did you experience any pain when using the VR?	0	93	7
● Do you want to use VR for learning in the future?	100	0	0
● Was it easy to operate the VR?	93	0	7
● When you used VR, did it feel like reality?	87	0	13
● Do you think you will be able to proceed with learning by using VR without teacher support next time?	33	40	27

A five-point questionnaire (Strongly Agree; Agree; Neither Agree nor Disagree; Disagree; Strongly Disagree) was administered to 15 students using HMD-VRC, and the sum of “Strongly Agree” and “Agree” was expressed as “Agree,” and similarly, the sum of “Strongly Disagree” and “Disagree” was expressed as “Disagree” as a percentage. “Neither Agree nor Disagree” was expressed as “Neither.” The top six items indicated that learning with HMD-VRC was favorably received

**Fig. 5:**
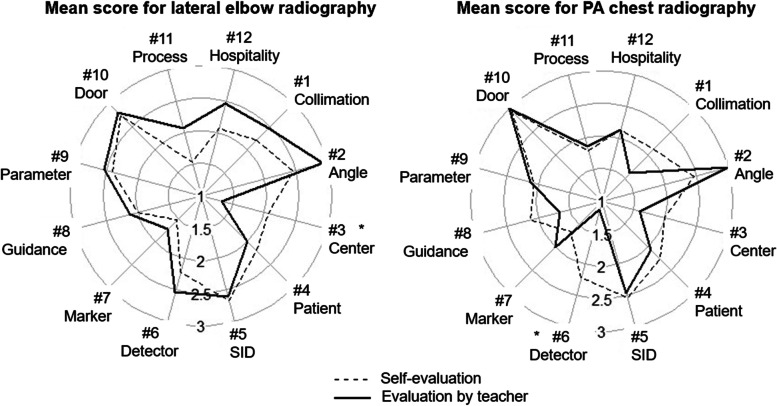
Evaluation of each skill by teachers and students themselves in the HMD-VRC group. The radar chart shows the average scores of the 12 items in the rubric table when the skills of the students using HMD-VRC were evaluated by the teachers and when the students performed a self-evaluation. The dotted line indicates self-evaluation, and the solid line indicates evaluation by the teachers. Each item corresponds to an item in Table 1. Each individual was scored on a four-point scale from 0 to 3. Self-ratings were significantly higher than teacher ratings in #3 for lateral elbow and #6 for PA chest radiography

Figure 5 shows the average scores for the 12 items in the rubric table when the students’ proficiency using HMD-VRC was evaluated by the teacher and when the students self-evaluated. The dotted line indicates self-evaluation, and the solid line indicates evaluation by the teachers. Each item corresponds to an item in Table 1. Each individual was scored on a four-point scale from 0 to 3. Self-evaluation was higher than objective evaluation by the teachers in “Location and centering of the X-ray beam” of lateral elbow radiography and in “Positioning the X-ray image detector” of PA chest radiography.

## Discussion

### Impact of HMD-VRC technical issues on proficiency

In this study, certain skills showed significantly lower proficiency in the HMD-VRC group than in the RP group, as shown in Fig. 4. Furthermore, differences in proficiency were observed depending on the targeted inspection site. First, the HMD-VRC group showed significantly lower ratings of “Location and centering of the X-ray beam” for lateral elbow radiography and “Positioning the X-ray image detector” for PA chest radiography than the RP group. These are typically positioned by touching the patient. Furthermore, “Palpation” has been reported as a technical issue for HMD-VRC [7]. This suggests that palpation to the human body has an impact on radiographic proficiency.

In addition, the HMD-VRC group was significantly less likely to use “Voice guidance” and show “Hospitality and concern for patient pain” in the PA chest than the RP group. These are the responses through interaction with the patient, including communication. Especially in PA chest radiography, there are a lot of instructions from radiologists to patients, so the radiologists should avoid actions to induce discomfort for patients. There are limitations to the patient interaction in VR at the current technological status. The VR environment was not sufficiently considered high fidelity compared to the use of real (standardized) patients. Fidelity is reported to be an important factor in simulation education in medicine [12]. Therefore, it was considered difficult to acquire skills such as, “Hospitality and concern for patient pain,” which are necessary in clinical practice. This is a major issue for education using VR systems. In the future, patient models with greater autonomy and responding appropriately with user actions would solve the problem of patient interaction required for radiography.

Furthermore, new issues were discovered in this study. In PA chest radiography, the HMD-VRC group was significantly lower than the RP group in “Process control and safety.” The HMD-VRC did not include a series of movements when the simulated patient entered or left the room, and the only movement was to adjust the examination position in front of the device. Usually there is some preparation before calling the patient. Therefore, we consider that the understanding of the radiographic procedure was not improved. It is also necessary to provide examples of the correct sequence of tasks within the VR. To increase learning effectiveness, it is necessary to increase the fidelity of the series of radiographic procedures. Every step of the process, from the time the patient enters the room until they leave, should be designed in such a way that the learner feels confident that they are really performing the procedure.

### Comparison with previous studies

Gunn et al. [10] reported that in their evaluation of proficiency using the rubric method, learning with VR and proficiency with hands-on training were nearly equivalent in all skills categories. However, as shown in Fig. 4, some skills’ evaluation was significantly lower in the HMD-VRC group than in the RP group. There are three differences between the report of Gunn et al. [10] and the present study. First, Gunn et al. used a VR environment with a PC monitor, while we used an HMD. Therefore, this is the first study to evaluate HMD-VRC proficiency through a rubric evaluation. Second, we evaluated inspection sites that require different radiographic skill than the researchers reported. PA chest radiography is the most common area of radiography and contains many important radiographic skills. Lateral elbow radiography is a typical joint radiography, in which the angle of incidence of radiation is adjusted by complex patient positioning to image the joint. Third, the rubric includes additional items required in clinical situations, such as communication. Because of these differences, this is the first study to evaluate HMD-VRC proficiency through rubric evaluation. We consider that these differences reveal the gap in acquired proficiency when using HMD-VRC.

O'Connor et al. [7] conducted a questionnaire for the students using HMD-VRC. They reported that the students responded that HMD-VRC contributed to their motivation to learn and improved their radiographic skills, which is consistent with this study. As shown in Additional file 1: Appendix 1, the results of the questionnaire survey on skills improvement showed no significant differences in all evaluated skills. This is contrary to the results shown in Fig. 4, where proficiency in several skills is lower. Arora et al. [13] report that some skill items are more difficult to self-evaluate than others, depending on the richness and variety in work experience. Therefore, when evaluating a new educational tool, it may not be possible to correctly evaluate learning effectiveness using only a questionnaire, and it is useful to evaluate it objectively using a rubric evaluation.

### Self-learning with HMD-VRC

The results of the questionnaire in Table 3 show that the students have a favorable impression of the HMD-VRC. However, 40% of the students expressed negative impressions about learning alone in the future. The main reason for this result would be that the HMD-VRC used in this study has no evaluation module for the students, who feel less confident in self-evaluation due to the early stages of learning and need objective evaluations.

As shown in Fig. 5, a comparison of proficiency evaluations by the teachers and the students themselves in this study revealed that some items were difficult to self-evaluate (“Location and centering of the X-ray beam” for lateral elbow radiography and “Positioning the X-ray image detector” for PA chest radiography). This is consistent with the report that self-evaluation tends to be significantly higher for some evaluation items when work experience is lower [13]. This suggests that self-learning requires timely and appropriate evaluation. These results suggest that the system’s ability to evaluate students is important for self-learning with HMD-VRC.

Conventional education does not allow students to learn at their own pace due to the use of irradiation equipment that requires safety control. However, HMD-VRC has the advantage that radiography training can be trained at the pace of one's own learning. This allows for further self-learning at home or during free time in class. Furthermore, it is expected that multiple uses of the HMD-VRC will enhance proficiency [14]. In the education of radiography skills, it is desirable to develop a system that enables more effective self-learning by taking countermeasures against the issues of HMD-VRC.

### Future direction

The results of this study confirm that the rubric method captures the characteristics of HMD-VRC training outcomes. The rubric method can be used to evaluate systems other than VR X-Ray used in this study. Previous studies have begun to solve the palpation and patient interaction issues of HMD-VRC [8, 15] and in the future, objective verification of the effectiveness of those systems is desired.

In this study, the operation was performed using a hand controller, but training with haptic presentation using a glove-type device is expected in the future [16]. We believe that this will increase the sense of fidelity when learning tasks that are essentially performed with the hands.

As for limitations of this experiment, the questionnaire item in Table 3, “Do you think you will be able to proceed with learning by using VR without teacher support next time?” used in this study was partially vague and it is possible that the questionnaire was not understood appropriately. Furthermore, the training time was too short to acquire the ability to practice in a clinical setting. The purpose of this study was to compare HMD-VRC with traditional education and to evaluate the impact of HMD-VRC issues. We determined that about one hour was needed to perform a series of tasks and understand the radiographic techniques. In the classroom setting, there are also time and location constraints. However, HMD-VRC had advantageous characteristics, in that it is not limited by time and location compared to practical training that uses physical equipment [17]. For future research, we are interested in the effects of the repeated use of HMD-VRC.

## Conclusions

This study suggests that HMD-VRC can be less effective than real-world training in radiographic techniques, which require palpation and patient interaction. However, the system used in this study has the limitation that it did not have the function to return evaluations to the learners. For effective training, it is important to objectively evaluate proficiency in the real world, even for HMD-VRC with new technologies, such as haptic presentation and VR patient interaction.

## Supplementary Information

**Additional file 1.**

## Data Availability

The datasets used and/or analyzed during the current study are available from the corresponding author on reasonable request.

## Abbreviations

HMD: Head Mounted Display
VR: Virtual Reality
HMD-VRC: HMD-based immersive VR Coaching systems
GPA: Grade Point Average
RP: Role-Play
PA: Postero-Anterior
