# Supplementary material for: Radiography education with VR using head mounted display: proficiency evaluation by rubric method
Source: BMC Med Educ. 2022 Jul 28;22:579. doi: 10.1186/s12909-022-03645-8 (PMC9331594; doi:10.1186/s12909-022-03645-8)
Supplement: Supplementary file 1 — Additional file 1. [file 12909_2022_3645_MOESM1_ESM.pdf]

Appendix 1. Questionnaire results for students who conducted the HMD-VRC group and the RP group.

| Question item | Group | SD | D | A | SA |
|---|---|---|---|---|---|
| Do you feel that your radiographic skills have improved? | HMD-VRC | 0 | 7 | 47 | 27 |
| | RP | 0 | 0 | 67 | 20 |
| Did you gain a better understanding of the anatomical structure of the human body at the radiographic areas you experienced this study? | HMD-VRC | 0 | 0 | 73 | 27 |
| | RP | 0 | 0 | 73 | 27 |
| Have you evaluated the images and gained a better understanding of acceptable imaging conditions? | HMD-VRC | 0 | 7 | 67 | 27 |
| | RP | 0 | 0 | 53 | 47 |
| Were you motivated to learn? | HMD-VRC | 0 | 0 | 33 | 53 |
| | RP | 0 | 0 | 53 | 47 |
| Has your interest in radiographic techniques increased? | HMD-VRC | 0 | 0 | 27 | 73 |
| | RP | 0 | 0 | 20 | 80 |
| Did you gain a better understanding of the differences in imaging conditions? | HMD-VRC | 0 | 0 | 53 | 47 |
| | RP | 0 | 0 | 80 | 20 |
| Were you satisfied with the learning process? | HMD-VRC | 0 | 0 | 40 | 53 |
| | RP | 0 | 0 | 53 | 47 |
| Did you gain a better understanding of the location of the X-ray beam? | HMD-VRC | 0 | 0 | 60 | 40 |
| | RP | 0 | 0 | 60 | 33 |
| Did you gain a better understanding of technical terms? | HMD-VRC | 0 | 0 | 73 | 20 |
| | RP | 0 | 0 | 80 | 7 |

| | | | | | |
|---|---|---|---|---|---|
| Did you gain a better understanding of the SID? | HMD-VRC | 0 | 0 | 67 | 33 |
| | RP | 0 | 0 | 53 | 47 |
| Did you gain a better understanding of the irradiation field? | HMD-VRC | 0 | 0 | 73 | 27 |
| | RP | 0 | 0 | 60 | 40 |
| Did you concentrate on your training? | HMD-VRC | 0 | 0 | 27 | 73 |
| | RP | 0 | 0 | 40 | 60 |
| Did you gain a better understanding of radiography safety management? | HMD-VRC | 0 | 0 | 67 | 33 |
| | RP | 0 | 0 | 73 | 20 |
| Did you gain a better understanding of patient positioning? | HMD-VRC | 0 | 0 | 67 | 33 |
| | RP | 0 | 0 | 53 | 40 |
| Did you gain a better understanding of the sequence of processes from preparation to imaging? | HMD-VRC | 0 | 0 | 67 | 27 |
| | RP | 0 | 0 | 40 | 60 |
| Did you gain a better understanding of how to use the equipment? | HMD-VRC | 0 | 0 | 40 | 53 |
| | RP | 0 | 0 | 47 | 53 |
| Did this study improve your motivation to learn more about learning radiography techniques in the future? | HMD-VRC | 0 | 0 | 33 | 67 |
| | RP | 0 | 0 | 13 | 87 |
| Are you interested in classes using VR? | HMD-VRC | 0 | 0 | 33 | 67 |
| | RP | 0 | 0 | 47 | 47 |

(%)

A five-point questionnaire (Strongly Agree; SA, Agree; A, Neither Agree nor Disagree, Disagree; D, Strongly Disagree; SD) was administered for students who conducted the HMD-VRC group (n=15) and RP group (n=15).There were no significant differences between the HMD-VRC group and the RP group on all questions.
